# Impact of race and ethnicity on presentation and outcomes of patients treated on rhabdomyosarcoma clinical trials: A report from the Children's Oncology Group

**DOI:** 10.1002/cam4.5921

**Published:** 2023-04-20

**Authors:** Senna R. Munnikhuysen, Princess A. Ekpo, Wei Xue, Zhengya Gao, Philip J. Lupo, Rajkumar Venkatramani, Christine M. Heske

**Affiliations:** ^1^ Pediatric Oncology Branch National Cancer Institute, National Institutes of Health Bethesda Maryland USA; ^2^ Department of Biostatistics, College of Public Health and Health Professions and College of Medicine University of Florida Gainesville Florida USA; ^3^ Division of Hematology/Oncology, Department of Pediatrics, Texas Children's Cancer Center Texas Children's Hospital, Baylor College of Medicine Houston Texas USA

## Abstract

**Background:**

Racial and ethnic disparities have been demonstrated in pediatric and adult cancers. However, there is no consensus on whether such disparities exist in the presentation, treatment, and outcome of patients with rhabdomyosarcoma (RMS).

**Methods:**

Patient information from the seven most recent RMS clinical trials was obtained from the Children's Oncology Group (COG). Chi‐squared analyses were used to compare patient, tumor, and treatment characteristics across racial and ethnic groups. Pairwise analyses comparing Non‐Hispanic Black (NHB) versus Non‐Hispanic White (NHW) racial groups and Hispanic versus NHW ethnic groups were conducted for significant characteristics. Kaplan–Meier method and Wilcoxon signed‐rank tests were performed to analyze outcomes.

**Results:**

In the overall cohort (*n* = 2157), patients' self‐identified race/ethnicity was: 0.4% American Indian/Alaska Native, 2.6% Asian, 12.6% Hispanic, 0.2% Native American/other Pacific Islander, 12.8% NHB, 61.9% NHW, and 9.6% unknown. Six characteristics differed by race/ethnicity: age, histology, IRS group, invasiveness, metastatic disease, and *FOXO1* fusion partner. Five were significant in pairwise comparisons: NHB patients were more likely to present at age ≥ 10 years and with invasive tumors than NHW patients; Hispanic patients were more likely to present with alveolar histology, metastatic disease, and IRS group IV disease than NHW patients. No differences were found in event free or overall survival of the entire cohort, in risk group‐based subset analyses, or among patients with high‐risk characteristics significant on pairwise analysis.

**Conclusions:**

While NHB and Hispanic patients enrolled in COG trials presented with higher risk features than NHW patients, there were no outcome differences by racial or ethnic group.

## Graphical abstract

How does the presentation and outcome of patients with rhabdomyosarcoma treated on clinical trials differ by race and ethnicity?

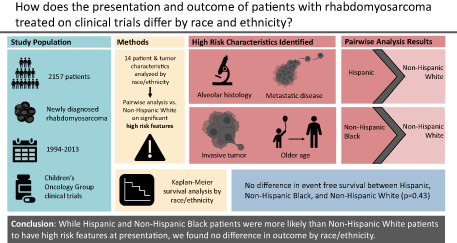

## 1 INTRODUCTION

For many cancers, patient race and ethnicity are recognized as important factors in terms of incidence and outcome.^1^, ^2^, ^3^ While racial and ethnic disparities have been widely demonstrated in some pediatric cancers,^4^, ^5^ there is currently no consensus about whether such disparities exist in the presentation, treatment, and outcome of rhabdomyosarcoma (RMS), the most common soft tissue sarcoma in children and adolescents.^6^

Several studies evaluating pediatric patients with solid tumors have shown that Non‐Hispanic Black (NHB) and Hispanic patients are more likely to present with high‐risk features than Non‐Hispanic White (NHW) patients.^7^, ^8^, ^9^, ^10^ The one study specifically evaluating children with RMS found that NHB patients were more likely than NHW patients to present with the high‐risk features of regional lymph node involvement, invasive tumors, and tumors >5 cm.^7^ These data are from studies completed over 25 years ago, and it is unknown whether these findings persist in the current era.

No study has investigated treatment differences by race or ethnicity specifically in children with RMS, but registry studies of children with soft tissue sarcoma (STS) have yielded conflicting results about the rates of surgical resection and use of radiation therapy by race or ethnicity. Two, studies have found that among patients with STS, NHB patients are less likely than NHW patients to undergo surgical resection,^9^, ^11^ while another found no difference.^12^ NHW patients had higher rates of radiation therapy than NHB patients in one SEER‐based study of pediatric patients with STS, but this result has not been replicated.^12^

Studies investigating the effect of race and ethnicity on outcomes in patients with pediatric solid tumors, sarcomas, or specifically RMS have produced opposing results. Some studies have shown that NHW patients experience improved survival compared to NHB and/or Hispanic patients,^8^, ^9^, ^11^, ^12^, ^13^, ^14^, ^15^, ^16^ yet others have not reported differences in outcome by race and/or ethnicity.^7^, ^17^ Furthermore, a number of studies that sought to identify prognostic factors for RMS report no data on race and/or ethnicity.^18^, ^19^, ^20^, ^21^, ^22^ The current Children's Oncology Group (COG) risk stratification does not include race or ethnicity as prognostic factors.^23^

Given conflicting data regarding the impact of race and ethnicity on clinical presentation, treatment, and outcomes of RMS, our study aims to describe the role of race/ethnicity on these factors in patients with pediatric‐type RMS through a retrospective analysis of data from recent interventional COG clinical trials for patients with newly diagnosed RMS.

## 2 METHODS

### 2.1 Study population

Patients with newly diagnosed RMS enrolled on one of seven previously reported COG studies (D9602, D9802, D9803, ARST0331, ARST0431, ARST0531, or ARST08P1) were included in this analysis.^24^, ^25^, ^26^, ^27^, ^28^, ^29^, ^30^ Studies were approved by institutional review boards (IRB) of participating institutions or Pediatric Central IRB of the National Cancer Institute (NCI). Informed consent/assent from patients and/or parents/guardians was obtained prior to enrollment.

### 2.2 COG data analysis

Two‐way cross tables were used to present distributions (frequency and percentage) of patient, tumor, and treatment characteristics by racial/ethnic groups. Distributions were compared using chi‐squared tests. Pairwise analyses comparing NHB versus NHW racial groups and Hispanic versus NHW ethnic groups were conducted for significant characteristics. The Kaplan–Meier method was used to estimate survival distributions and the Peto–Peto method was used to estimate the standard error of the Kaplan–Meier estimate. Log–rank tests were performed to compare event‐free survival (EFS) and overall survival (OS) by race/ethnicity.

### 2.3 SEER data analysis

To contextualize representation of patients on COG trials, we extracted incidence data between 1994 and 2013 from the NCI‐funded Surveillance, Epidemiology, and End Results (SEER) Program using the SEER*Stat software (version 8.4.0.1), including data submitted from 13 registries through November 2020. SEER 13 was used to be consistent with the majority of previous population‐based estimates of RMS incidence. RMS subtypes included embryonal, spindle cell, alveolar, mixed type, and NOS. Race/ethnicity classification was based on SEER race and origin recode. Data were extracted from patients aged 0–45 years. Distribution of race/ethnicity from the SEER and COG cohorts were compared using Chi‐squared tests.

## 3 RESULTS

### 3.1 Clinical presentation

Between 1994 and 2013 a total of 2157 patients up to 45 years of age with newly diagnosed RMS enrolled on COG studies. Fifty‐six patients (2.6%) were identified as Asian, 271 (12.6%) identified as Hispanic, 275 (12.8%) identified as NHB, and 1335 (61.9%) identified as NHW. Eight patients (0.4%) were identified as American Indian or Alaska Native, and 4 (0.2%) identified as Native American or other Pacific Islander; these groups were combined into a cohort designated “Other” due to the small number of patients. Representing 0.6% of the total study population, the “Other” cohort was excluded from statistical analysis due to insufficient sample size. Two hundred and eight patients (9.6%) had unknown race/ethnicity and were also excluded from analysis as no further information about their demographics was available. Table S1 includes the complete list of patient and tumor characteristics by race/ethnicity. Table S2 includes the racial breakdown of patients with Hispanic ethnicity.

**FIGURE 1:**
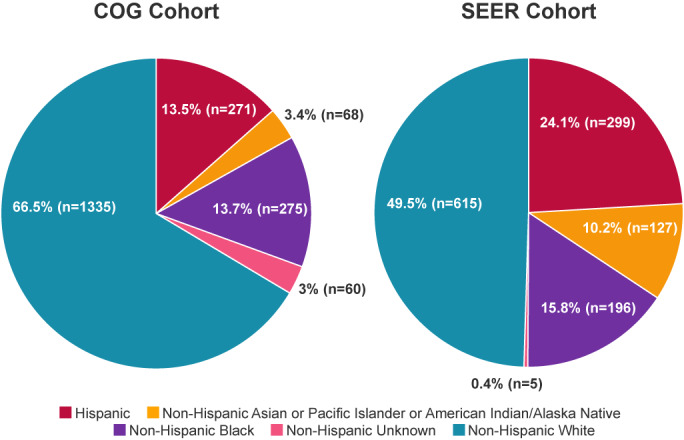
Comparison of race/ethnicity distribution between COG cohort and SEER cohort. Pie chart of the racial/ethnic distribution of patients in the Children's Oncology Group cohort and the SEER cohort including Hispanic (red), Non‐Hispanic Asian or Pacific Islander or American Indian/Alaska Native (orange), Non‐Hispanic Black (purple), Non‐Hispanic Unknown (pink), and Non‐Hispanic White (teal). Unknown race were removed from COG dataset prior to comparison. *P*‐value <0.0001 obtained using Chi‐squared test.

Data extracted from the SEER database regarding patient race/ethnicity for 1242 patients of the same ages diagnosed during the same time‐period as the COG cohort is presented in Figure 1 and Table S3. A comparison of the two cohorts indicated that the racial and ethnic distribution was different (*p* < 0.0001) with the COG cohort including a larger proportion of NHW patients and a smaller proportion of Hispanic patients than the SEER cohort (Table S4).

To identify differences in the presenting characteristics of patients and tumors by race/ethnicity, univariable analysis was performed on 14 patient and tumor factors for the four largest patient groups: Asian, Hispanic, NHB, and NHW (Table 1). Six factors were found to be significantly different when analyzed by racial and ethnic group: age at presentation (*p* = 0.0037), histology (*p* = 0.0446), Intergroup RMS Study (IRS) group (*p* = 0.0287), tumor invasiveness (*p* = 0.0347), metastatic disease (*p* = 0.0101), and *FOXO1* fusion partner (*PAX3* or *PAX7*) in fusion positive tumors (*p* = 0.0131). Sex, IRS stage, tumor size, nodal status, primary site, metastatic site, and *FOXO1* fusion status (positive or negative) were not significantly different.

**TABLE 1:** Univariable analysis of effect of race/ethnicity on clinical presentation of RMS.

Characteristic	Race/ethnicity								*p*‐Value*
	Asian		Hispanic		Non‐Hispanic Black		Non‐Hispanic White		
	*N*	%	*N*	%	*N*	%	*N*	%	
Overall No.	56	2.9	271	14.0	275	14.2	1335	68.9	
Age									**0.0037**
<1 year	1	1.1	19	21.6	9	10.2	59	67.0	
1–9 year	36	3.2	163	14.4	137	12.1	799	70.4	
≥10 year	19	2.7	89	12.5	129	18.1	477	66.8	
Sex									0.3798
Female	17	2.2	105	13.7	117	15.2	529	68.9	
Male	39	3.3	166	14.2	158	13.5	806	68.9	
RMS histology									**0.0446**
Alveolar	20	3.3	105	17.1	93	15.1	397	64.6	
Embryonal or Botryoid	31	2.9	127	11.9	155	14.6	752	70.6	
Spindle cell	3	1.9	21	13.1	13	8.1	123	76.9	
Mixed or NOS	2	2.4	14	16.9	12	14.5	55	66.3	
IRS group									**0.0287**
I	11	4.0	36	13.2	39	14.3	187	68.5	
II	3	1.0	32	10.7	43	14.4	220	73.8	
III	31	3.2	129	13.3	131	13.5	679	70.0	
IV	11	2.8	74	18.8	61	15.5	247	62.8	
Tumor invasiveness									**0.0347**
T1, Not invasive	30	3.0	130	12.9	126	12.5	723	71.7	
T2, Invasive	26	2.8	141	15.3	148	16.0	608	65.9	
IRS stage									0.0782
1	16	2.4	80	11.9	93	13.8	484	71.9	
2	12	4.0	39	13.0	46	15.4	202	67.6	
3	17	3.0	78	13.7	74	13.0	400	70.4	
4	11	2.8	74	18.8	61	15.5	247	62.7	
Tumor size									0.9168
≤5 cm	30	3.1	133	13.7	135	13.9	676	69.4	
>5 cm	26	2.8	135	14.6	131	14.2	633	68.4	
Nodal status									
Nodal clinical									0.0531
N0	40	2.8	187	13.2	189	13.3	1001	70.6	
N1	16	3.3	80	16.5	79	16.3	310	63.9	
Nodal path									0.3863
N0	16	3.7	65	14.8	68	15.5	289	66.0	
N1	7	4.7	26	17.6	29	19.6	86	58.1	
Metastatic disease									**0.0101**
Present	11	2.8	74	18.9	60	15.3	247	63.0	
Absent	45	2.9	197	12.8	214	13.9	1088	70.5	
Primary site									0.2704
Favorable	19	2.7	86	12.1	106	14.9	502	70.4	
Unfavorable	37	3.0	185	15.1	169	13.8	833	68.1	
Metastatic site									0.7804
Lungs	6	3.9	27	17.4	21	13.5	101	65.2	
Bone or bone marrow	5	2.3	42	19.5	33	15.3	135	62.8	
Distant lymph nodes	5	3.4	26	17.9	25	17.2	89	61.4	
Soft tissue	7	7.0	20	20.0	15	15.0	58	58.0	
*FOXO1* fusion status									0.1623
+	12	3.3	62	17.1	61	16.8	228	62.8	
−	6	3.5	31	18.2	16	9.4	117	68.8	
*PAX* fusion partner (for fusion +)									**0.0131**
*PAX3*	6	2.3	43	16.7	48	18.6	161	62.4	
*PAX7*	6	11.3	6	11.3	7	13.2	34	64.2	

*Note*: *p*‐Value <0.05 was considered significant are in bold.

Abbreviations: IRS, Intergroup Rhabdomyosarcoma Study; NOS, not otherwise specified; RMS, rhabdomyosarcoma.

* *p*‐value was obtained using Chi‐squared test.

To further evaluate the six significant factors identified on univariable analysis, pairwise comparisons were performed between the two largest racial groups, NHB and NHW, and the two largest ethnic groups, Hispanic and NHW (Table 2). This analysis demonstrated that age of presentation, categorized using the standard risk‐based age cut‐offs of <1 year, 1–9 years, or ≥ 10 years, was significantly different between NHB and NHW patients (*p* = 0.0022). Rates of presentation in infancy were similar between these groups (3.3% for NHB and 4.4% for NHW patients). However, while NHW patients were more likely to present at ages between 1–9 years than ≥10 years (59.9% vs. 35.7%), NHB patients were nearly equally likely to present in these two age groups (49.8% vs. 46.9%) (Table S1). Tumor invasiveness was also found to be significantly different between the NHB and NHW groups (*p* = 0.0118). Among NHB patients, 54% had invasive tumors at presentation while among NHW, only 45.7% did (Table S1). By pairwise analysis of NHB and NHW patients, histology, IRS group, the presence of metastatic disease, and *FOXO1* fusion partner were not significantly different.

**TABLE 2:** Pairwise analysis between Non‐Hispanic Black versus Non‐Hispanic White patients and Hispanic versus Non‐Hispanic White patients for age group, RMS histology, Intergroup Rhabdomyosarcoma Study (IRS) group, tumor invasiveness, metastatic disease, and *FOXO1* fusion partner (*PAX3* or *PAX7*).

*p*‐Value*	NHB vs. NHW	H vs. NHW
Age	**0.0022**	0.1611
RMS histology	0.0814	**0.0135**
IRS group	0.5336	**0.0061**
Invasiveness	**0.0118**	0.0562
Metastatic disease	0.2024	**0.0010**
*FOXO1* fusion partner (for fusion +)	0.4049	0.3802

*Note*: *p*‐Value <0.05 was considered significant are in bold.

Abbreviations: H, Hispanic; IRS, Intergroup Rhabdomyosarcoma Study; NBH, Non‐Hispanic Black; NHW, Non‐Hispanic White.

* *p*‐value was obtained using Chi‐squared test.

Pairwise analysis of Hispanic and NHW ethnic groups for the same six factors identified and revealed significant differences in histology (*p* = 0.0135), IRS group (*p* = 0.0061) and metastatic disease (*p* = 0.0010) between these populations. Hispanic patients were more likely to present with alveolar histology (38.7% vs. 29.7%) and IRS group IV or metastatic disease (27.3% vs. 18.5%) than NHW patients (Table S1). Age at presentation, tumor invasiveness, and *FOXO1* fusion partner were not significantly different between Hispanic and NHW patients.

### 3.2 Treatment

Chi‐squared tests were performed to evaluate for differences in the extent of surgical resection in localized patients (represented by IRS groups I‐III) and use of radiation therapy between Asian, Hispanic, NHB, and NHW patients. No significant difference in either treatment factor was noted between these racial and ethnic groups (Table S5).

### 3.3 Outcome

**FIGURE 2:**
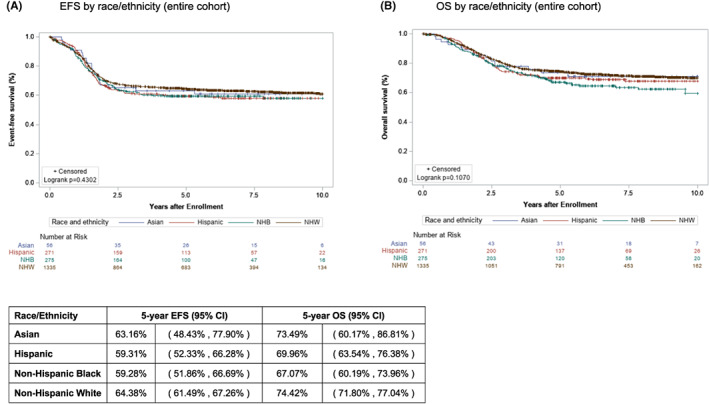
Event‐free survival (EFS) and overall survival (OS) of total cohort by race/ethnicity. Kaplan–Meier curves representing (A) EFS and (B) OS for the complete population of Asian (blue), Hispanic (red), Non‐Hispanic Black (green), and Non‐Hispanic White (brown) patients with rhabdomyosarcoma treated on Children's Oncology Group studies between 1994 and 2013.

**FIGURE 3:**
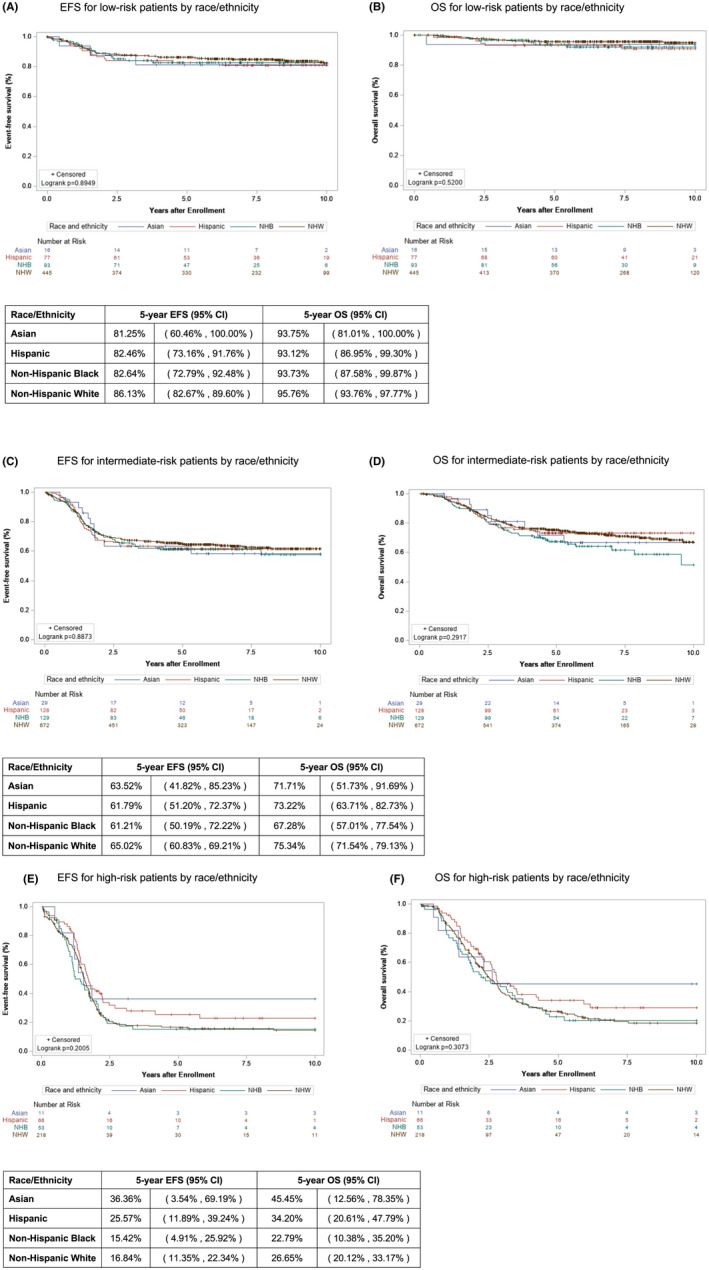
Event‐free survival (EFS) and overall survival (OS) of patients in each rhabdomyosarcoma risk group by race/ethnicity. Kaplan–Meier curves representing (A) EFS and (B) OS for Asian (blue), Hispanic (red), Non‐Hispanic Black (green), and Non‐Hispanic White (brown) patients with low‐risk rhabdomyosarcoma (RMS) treated on protocols D9603 and ARST0331. Kaplan–Meier curves representing (C) EFS and (D) OS for Asian (blue), Hispanic (red), Non‐Hispanic Black (green), and Non‐Hispanic White (brown) patients with intermediate‐risk RMS treated on protocols D9803 and ARST0531. Kaplan–Meier curves representing (E) EFS and (F) OS for Asian (blue), Hispanic (red), Non‐Hispanic Black (green), and Non‐Hispanic White (brown) patients with high‐risk RMS treated on protocols D9802, ARST08P1 and ARST0431.

Differences in outcome by race/ethnicity were evaluated using Kaplan–Meier curves for EFS and OS for Asian, Hispanic, NHB, and NHW patients. Relative outcomes of all patients and of patients by RMS risk group (low, intermediate, and high) were evaluated. For the overall cohort, no significant difference was found for EFS or OS between the racial and ethnic groups (Figure 2A,B). Similarly, no differences were found in EFS or OS for patients with low‐, intermediate‐, or high‐risk RMS (Figure 3A–F).

**FIGURE 4:**
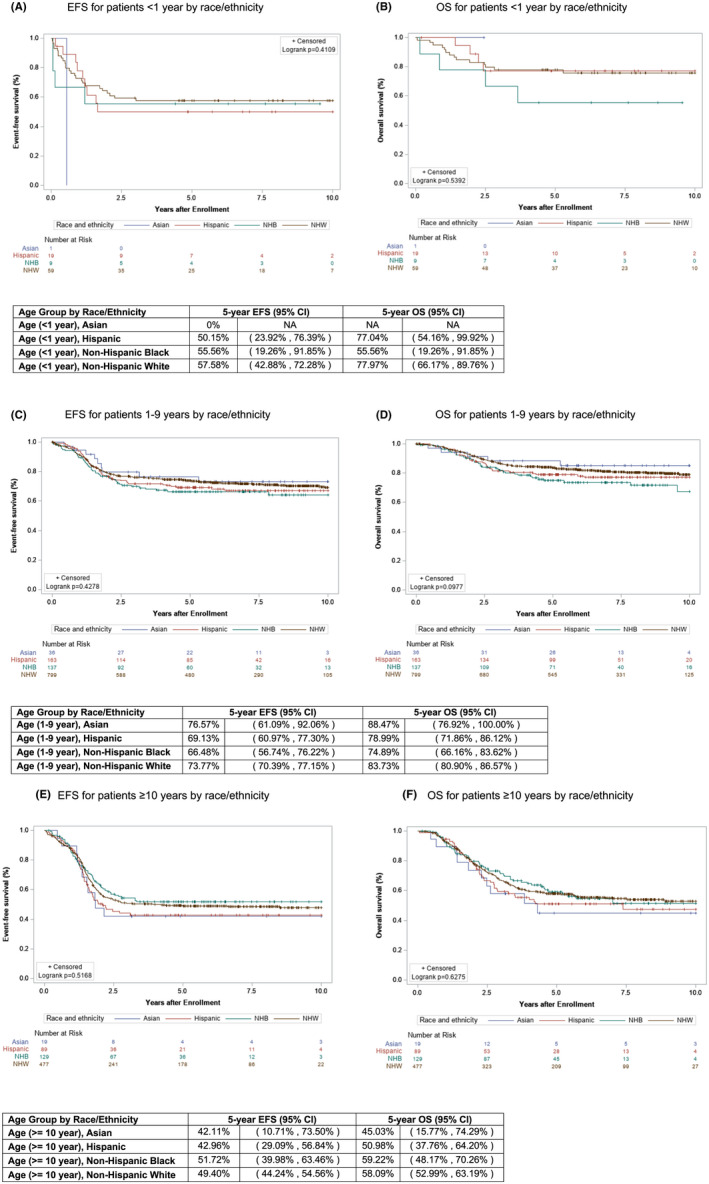
Event‐free survival (EFS) and overall survival (OS) of patients in each age category by race/ethnicity. Kaplan–Meier curves representing (A) EFS and (B) OS for Asian (blue), Hispanic (red), Non‐Hispanic Black (green), and Non‐Hispanic White (brown) patients aged <1 year. Kaplan–Meier curves representing (C) EFS and (D) OS for Asian (blue), Hispanic (red), Non‐Hispanic Black (green), and Non‐Hispanic White (brown) patients aged 1–9 years. Kaplan–Meier curves representing (E) EFS and (F) OS for Asian (blue), Hispanic (red), Non‐Hispanic Black (green), and Non‐Hispanic White (brown) patients aged ≥10 years.

**FIGURE 5:**
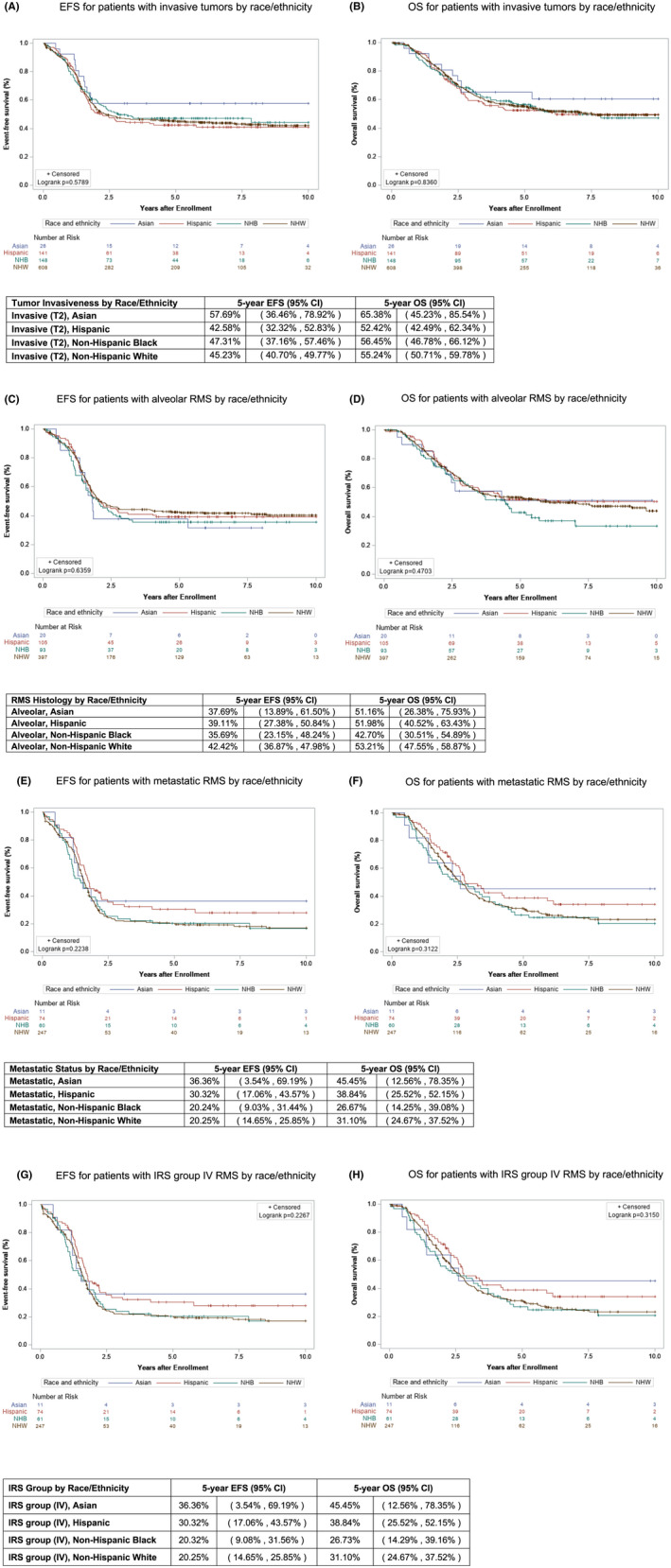
Event‐free survival (EFS) and overall survival (OS) of patients with additional high‐risk features by race/ethnicity. Kaplan–Meier curves representing (A) EFS and (B) OS for Asian (blue), Hispanic (red), Non‐Hispanic Black (green), and Non‐Hispanic White (brown) patients with invasive (T2) tumors. Kaplan–Meier curves representing (C) EFS and (D) OS for Asian (blue), Hispanic (red), Non‐Hispanic Black (green), and Non‐Hispanic White (brown) patients with alveolar histology. Kaplan–Meier curves representing (E) EFS and (F) OS for Asian (blue), Hispanic (red), Non‐Hispanic Black (green), and Non‐Hispanic White (brown) patients with metastatic disease. Kaplan–Meier curves representing (G) EFS and (H) OS for Asian (blue), Hispanic (red), Non‐Hispanic Black (green), and Non‐Hispanic White (brown) patients with IRS group IV disease.

To examine whether differences in presenting features impacted outcomes, Kaplan–Meier curves were also created for the features that had emerged as significantly different by race/ethnicity in the univariable analysis and pairwise analyses: age, histology, invasiveness, metastatic disease, and IRS group. Each age cohort (<1 year, 1–9 years, ≥10 years) was evaluated for differences in EFS and OS by race/ethnicity, and no significant survival differences were found for any of the three age cohorts (Figure 4A–F). Similarly, no differences were found between ethnic and racial groups for EFS or OS in patients with the characteristics that were found to be significant on pairwise analysis including invasive tumors, which extend into surrounding tissue (T2), alveolar histology, metastatic disease, or IRS group IV disease (Figure 5A–H).

## 4 DISCUSSION

The aim of this study was to describe the impact of race and ethnicity on the presentation, treatment, and outcome of patients with newly diagnosed RMS treated in recent COG clinical trials. This is the largest and most comprehensive study of the effects of race and/or ethnicity in RMS clinical trial patients in the modern treatment era. In contrast to many previous studies using population‐based registry data, this dataset included greater detail for factors related to clinical presentation such as IRS group, invasiveness, IRS stage, and tumor molecular features. This permitted analysis of outcomes for patients by risk group, which is critical in interpreting and contextualizing survival of RMS patients.

A key finding of this study is that differences in several presenting features of RMS exist between racial and ethnic groups, with NHB and Hispanic patients more likely to present with high‐risk factors, compared to NHW. Specifically, NHB patients were more likely to present at older age than NHW patients, which is a known adverse prognostic factor for RMS.^18^, ^19^, ^31^ The cause of this difference is unknown and has not previously been reported in the literature. Since ancestry data were not collected, no biologic or genetic explanation can be speculated. In addition, NHB patients were more likely than NHW patients to present with invasive tumors (T2 status), another poor prognostic factor.^18^, ^31^, ^32^, ^33^, ^34^ This finding is consistent with the previous report from a large consortium study of children treated on IRS III, IV‐pilot, and IV, which reported that non‐White patients were more likely to have invasive tumors at diagnosis than White patients.^7^ Importantly, it remains unknown whether invasive disease is related to underlying RMS tumor biology, whether it is a function of time‐related tumor progression, or both. Acknowledging this limitation, several possible explanations for this finding exist. While there may be a biological or genetic explanation, our lack of ancestry data prohibits us from evaluating this. Data capturing socioeconomic status and other social determinants of health were not collected, so we cannot evaluate for the possible confounding effect between race and access to care in this study. Finally, without data regarding time from symptom onset to diagnosis for NHB patients, we cannot evaluate the potential role of delayed diagnosis due to bias either at the provider, health system, or patient level. Multiple studies have found evidence of implicit pro‐White bias that affects pediatric providers' medical decisions, potentially leading to diagnostic delays.^35^, ^36^

Analysis of the two largest ethnic groups, Hispanic and NHW patients, also revealed differences in disease presentation. Hispanic patients were more likely to present with alveolar histology and metastatic or group IV disease than NHW patients. Among patients with localized RMS, alveolar histology is an adverse prognostic factor,^18^ while the presence of metastatic or group IV disease is the most important adverse prognostic factor for both newly diagnosed and relapsed RMS,^37^, ^38^ suggesting that Hispanic patients with RMS are at higher risk of poor outcomes. As with tumor invasiveness, it is not known whether the presence of metastases at diagnosis reflects disease biology or time‐related tumor progression, and study limitations prevent these evaluations.

Despite our findings that NHB and Hispanic patients are more likely than NHW patients to present with higher‐risk features, we found no evidence of differences in EFS or OS by race or ethnicity. This finding held true in the overall cohort and across each risk group, as well as within subgroup analyses of patients with specific high‐risk clinical features (older age, alveolar histology, invasive tumors, group IV, or metastatic disease). Three previous studies have evaluated how race and/or ethnicity affect outcome in pediatric RMS patients, and the results are conflicting. In the study of IRS clinical trials, there was no evidence of a difference in 5‐year failure free survival by race or ethnicity.^7^ In contrast, a recent population‐based study using US Cancer Statistics and National Program of Cancer Registries data found that among children with RMS, NHW patients had higher 5 year relative survival compared to NHB patients.^16^ Similarly, a recent SEER‐based study of children with RMS under 10 years of age reported that Hispanic ethnicity was independently associated with worse survival.^15^ Broader studies of pediatric solid tumor or sarcoma patients have yielded generally similar results. A SEER based study of pediatric cancer patients from 2000 until 2011 reported that Black patients had a significantly higher hazard of death compared to White patients in most pediatric cancers, including in the RMS cohort, although no differences were found between outcomes of the Hispanic and Non‐Hispanic RMS patients.^14^ Another SEER‐based study of pediatric patients with extracranial solid tumors from 1985 until 2005 reported a higher risk of death for Hispanic patients with RMS, compared to Non‐Hispanic patients with RMS, but did not find a higher risk of death for NHB patients with RMS.^13^ A third SEER‐based study demonstrated that White patients with RMS enjoyed greater survival improvements over the period of 1975 until 1999 than their non‐White counterparts.^39^ Two additional SEER‐based studies of patients with extremity (1973–2013) or chest wall (1998–2013) sarcomas, found that NHB patients were found to have inferior OS, compared to other groups, although non‐RMS and RMS patients were not analyzed separately.^9^, ^11^ A study of patients with pediatric non‐CNS solid tumors from the Texas Cancer Registry (1995–2009) similarly found that overall, NHB patients had worse survival probability than NHW patients, although no subgroup analyses by disease were performed.^8^ Importantly, in both studies that exclusively included clinical trial participants with RMS, ours and the IRS study, NHB and/or Hispanic patients presented with higher risk features than NHW patients, but there were no differences detected in outcome by race or ethnicity. This stands in contrast to the population‐based studies that repeatedly found disparities between survival outcomes of NHB or Hispanic patients and White or NHW patients, and included patients treated outside of clinical trials. One explanation for why population‐based studies have reported differences in survival by race and/or ethnicity while the studies of clinical trial participants have not is because clinical trial enrollment may provide a more standardized clinical approach. Indeed, in our limited evaluation of treatment, we found no differences in extent of surgical resection or use of radiation therapy by race or ethnicity. This may reflect how standardization of treatment on clinical trials facilitates equitable care and minimizes the impact of implicit bias. The outcome findings of our study indicate that despite presenting with higher risk clinical features at diagnosis, NHB and Hispanic RMS patients who are treated on clinical trials have outcomes that are more similar to their NHW counterparts than those not treated on clinical trials. This suggests that NHB and Hispanic patients with RMS may experience a survival benefit from being treated on clinical trials. An alternative explanation is that through selection bias, NHB and Hispanic clinical trial participants have a lower relative risk at diagnosis compared to those not treated on clinical trials. The ability to ascertain an individual patient's risk at diagnosis from registry data is limited, making this possibility difficult to evaluate.

Finally, comparing the racial and ethnic breakdown of the COG cohort to the SEER cohort we found that the COG cohort includes a significantly higher proportion of NHW patients. This is consistent with numerous studies reporting lower clinical trial enrollment for racial and ethnic minorities, including for pediatric oncology trials.^40^, ^41^, ^42^, ^43^, ^44^ While data from previously published studies suggest that clinical trials conducted by COG are largely accessible to patients of all races and ethnicities,^42^, ^45^, ^46^, ^47^ our data suggest that patients from ethnic and racial minority groups remain underrepresented in RMS trials. In addition, the COG cohort included a high number of participants who identified as unknown race (9.6%) compared to that of the SEER cohort (0.4%). One possible explanation is that for some patients enrolled on COG trials, their self‐identified races and ethnicities were not included as options. For example, there was no option for patients to choose multiple races or a multiracial category which, based on 2020 United States (US) census data, comprises 10.2% of Americans.^48^ Additionally, 33.6% of patients with Hispanic ethnicity did not identify any race, and consequently were categorized as unknown race. This may also be the result of inadequate options, as 2020 US census data demonstrated that 32.7% of Hispanic Americans identified as more than one race and 42.2% of Hispanic Americans identified as “Some Other Race”.^48^, ^49^ To more adequately capture data on race and ethnicity in future studies, we propose the use of standard data collection methods that include an option to select more than one race and/or ethnicity and an option to write in a more specific identifier when “Other” is selected.

In conclusion, our study evaluated the effect of race/ethnicity on the presentation, treatment, and outcome of newly diagnosed RMS patients treated on recent COG clinical trials. We found that NHB and Hispanic patients were more likely than NHW patients to present with higher risk features but that there were no differences in survival by race or ethnicity, in contrast to the findings of most population‐based studies of patients not exclusively treated on clinical trials. This suggests there may be a survival benefit for NHB and Hispanic patients who are treated on clinical trials and further underscores the importance of improving representation of minority and minoritized populations in clinical trials.

## AUTHOR CONTRIBUTIONS

**Senna Rae Munnikhuysen:** Conceptualization (equal); project administration (equal); writing – original draft (equal); writing – review and editing (equal). **Princess A. Ekpo:** Conceptualization (equal); writing – original draft (equal); writing – review and editing (equal). **Wei Xue:** Data curation (equal); formal analysis (equal); writing – original draft (equal). **Zhengya Gao:** Data curation (equal); formal analysis (equal); writing – original draft (equal). **Philip J. Lupo:** Writing – review and editing (equal). **Rajkumar Venkatramani:** Conceptualization (equal); supervision (equal); writing – review and editing (equal). **Christine Marie Heske:** Conceptualization (equal); project administration (equal); writing – original draft (equal); writing – review and editing (equal).

## FUNDING INFORMATION

This work was supported in part by the Summer's Way Foundation, Maddie's Promise Foundation, Sebastian Strong Foundation, Friends of TJ Foundation, St. Baldrick's Foundation, the National Cancer Institute at the National Institutes of Health (U10CA180886, U10CA180899, U10CA098543, and U10CA098413), and the Intramural Research Program of the National Institutes of Health.

## CONFLICT OF INTEREST STATEMENT

The authors have no conflicts of interest to disclose.

## ROLE OF THE FUNDER

The funder did not play a role in the design of the study; the collection, analysis, and interpretation of the data; the writing of the manuscript; and the decision to submit the manuscript for publication.

## PRIOR PRESENTATION

This study has been previously presented in part:Ekpo P, Munnikhuysen SR, Xue W, Gao Z, Venkatramani R, Heske CM. Racial and ethnic differences in presentation and clinical outcomes for pediatric rhabdomyosarcoma (RMS). Poster presentation and poster discussion at American Society of Clinical Oncology Annual Meeting [Abstract 10,017; Poster 232]. June 2022.

## Supporting information


Supporting information S1.

## Data Availability

The data that support the findings of this study are available from Children's Oncology Group. Restrictions apply to the availability of these data, which were used under license for this study. Data are available from the authors with the permission of Children's Oncology Group.
