# Supplementary material for: Impact of race and ethnicity on presentation and outcomes of patients treated on rhabdomyosarcoma clinical trials: A report from the Children's Oncology Group
Source: Cancer Med. 2023 Apr 20;12(11):12777–91. doi: 10.1002/cam4.5921 (PMC10278507; doi:10.1002/cam4.5921)
Supplement: Supplementary file 1 — Supporting information S1. [file CAM4-12-12777-s001.docx]

**Supplemental Material**

**Supplemental Table S1:** Characteristics of all patients by race/ethnicity

| **Characteristic** | **Entire Cohort** | | **Race/Ethnicity** | | | | | | | | | | | |
| --- | --- | --- | --- | --- | --- | --- | --- | --- | --- | --- | --- | --- | --- | --- |
|  |  | | **Asian** | | **Hispanic** | | **Non-Hispanic Black** | | **Non-Hispanic White** | | **Other^[[1]](#footnote-1)^** | | **Unknown** | |
|  | **N** | **%** | **N** | **%** | **N** | **%** | **N** | **%** | **N** | **%** | **N** | **%** | **N** | **%** |
| Overall No. | 2157 | 100 | 56 | 2.6 | 271 | 12.6 | 275 | 12.8 | 1335 | 61.9 | 12 | 0.6 | 208 | 9.6 |
| **Age** | | | | | | | | | | | | | | |
| <1 year | 95 | 4.4 | 1 | 1.8 | 19 | 7.0 | 9 | 3.3 | 59 | 4.4 | 0 | 0 | 7 | 3.4 |
| 1-9 year | 1271 | 58.9 | 36 | 64.3 | 163 | 60.1 | 137 | 49.8 | 799 | 59.9 | 10 | 83.3 | 126 | 60.6 |
| ≥10 year | 791 | 36.7 | 19 | 33.9 | 89 | 32.8 | 129 | 46.9 | 477 | 35.7 | 2 | 16.7 | 75 | 36.1 |
| **Sex** | | | | | | | | | | | | | | |
| Female | 858 | 39.8 | 17 | 30.4 | 105 | 38.7 | 117 | 42.5 | 529 | 39.6 | 7 | 58.3 | 83 | 39.9 |
| Male | 1299 | 60.2 | 39 | 69.6 | 166 | 61.3 | 158 | 57.5 | 806 | 60.4 | 5 | 41.7 | 125 | 60.1 |
| **RMS histology** | | | | | | | | | | | | | | |
| Alveolar | 688 | 31.9 | 20 | 35.7 | 105 | 38.7 | 93 | 33.8 | 397 | 29.7 | 3 | 25.0 | 70 | 33.7 |
| Embryonal or BRMS | 1184 | 54.9 | 31 | 55.4 | 127 | 46.8 | 155 | 56.4 | 752 | 56.4 | 8 | 66.6 | 111 | 53.4 |
| Spindle Cell | 180 | 8.3 | 3 | 5.4 | 21 | 7.7 | 13 | 4.7 | 123 | 9.2 | 1 | 8.3 | 19 | 9.1 |
| Mixed RMS | 20 | 0.9 | 1 | 1.8 | 2 | 0.7 | 3 | 1.1 | 12 | 0.9 | 0 | 0 | 2 | 1.0 |
| NOS | 71 | 3.3 | 1 | 1.8 | 12 | 4.4 | 9 | 3.3 | 43 | 3.2 | 0 | 0 | 6 | 2.9 |
| Other | 12 | 0.6 | 0 | 0 | 4 | 1.5 | 1 | 0.4 | 7 | 0.5 | 0 | 0 | 0 | 0 |
| Unknown | 2 | 0.1 | 0 | 0 | 0 | 0 | 1 | 0.4 | 1 | 0.1 | 0 | 0 | 0 | 0 |
| **IRS group** | | | | | | | | | | | | | | |
| I | 319 | 14.8 | 11 | 19.6 | 36 | 13.3 | 39 | 14.2 | 187 | 14.0 | 0 | 0 | 46 | 22.1 |
| II | 337 | 15.6 | 3 | 5.4 | 32 | 11.8 | 43 | 15.6 | 220 | 16.5 | 1 | 8.3 | 38 | 18.3 |
| III | 1068 | 49.5 | 31 | 55.4 | 129 | 47.6 | 131 | 47.6 | 679 | 50.9 | 8 | 66.7 | 90 | 43.3 |
| IV | 430 | 19.9 | 11 | 19.6 | 74 | 27.3 | 61 | 22.2 | 247 | 18.5 | 3 | 25.0 | 34 | 16.3 |
| Unknown | 3 | 0.1 | 0 | 0 | 0 | 0 | 1 | 0.4 | 2 | 0.1 | 0 | 0 | 0 | 0 |
| **Tumor invasiveness** | | | | | | | | | | | | | | |
| T1, Not Invasive | 1148 | 53.2 | 30 | 53.6 | 130 | 48.0 | 126 | 45.8 | 723 | 54.2 | 6 | 50.0 | 133 | 63.9 |
| T2, Invasive | 1004 | 46.5 | 26 | 46.4 | 141 | 52.0 | 148 | 53.8 | 608 | 45.5 | 6 | 50.0 | 75 | 36.1 |
| Unknown | 5 | 0.2 | 0 | 0 | 0 | 0 | 1 | 0.4 | 4 | 0.3 | 0 | 0 | 0 | 0 |
| **IRS Stage** | | | | | | | | | | | | | | |
| 1 | 780 | 36.2 | 16 | 28.6 | 80 | 29.5 | 93 | 33.8 | 484 | 36.3 | 2 | 16.7 | 105 | 50.5 |
| 2 | 330 | 15.3 | 12 | 21.4 | 39 | 14.4 | 46 | 16.7 | 202 | 15.1 | 2 | 16.7 | 29 | 13.9 |
| 3 | 613 | 28.4 | 17 | 30.4 | 78 | 28.8 | 74 | 26.9 | 400 | 30.0 | 5 | 41.7 | 40 | 19.2 |
| 4 | 431 | 20 | 11 | 19.6 | 74 | 27.3 | 61 | 22.2 | 247 | 18.5 | 3 | 25.0 | 34 | 16.3 |
| Unknown | 3 | 0.1 | 0 | 0 | 0 | 0 | 1 | 0.4 | 2 | 0.1 | 0 | 0 | 0 | 0 |
| **Tumor size** | | | | | | | | | | | | | | |
| ≤5cm | 1101 | 51 | 30 | 53.6 | 133 | 49.1 | 135 | 49.1 | 676 | 50.6 | 5 | 41.7 | 122 | 58.7 |
| >5cm | 1016 | 47.1 | 26 | 46.4 | 135 | 49.8 | 131 | 47.6 | 633 | 47.4 | 7 | 58.3 | 84 | 40.4 |
| Unknown | 40 | 1.9 | 0 | 0 | 3 | 1.1 | 9 | 3.3 | 26 | 1.9 | 0 | 0 | 2 | 1.0 |
| **Nodal status** | | | | | | | | | | | | | | |
| ***Clinical*** |  | | | | | | | | | | | | | |
| N0 | 1588 | 73.6 | 40 | 71.4 | 187 | 69.0 | 189 | 68.7 | 1001 | 75.0 | 11 | 91.7 | 160 | 76.9 |
| N1 | 530 | 24.6 | 16 | 28.6 | 80 | 29.5 | 79 | 28.7 | 310 | 23.2 | 1 | 8.3 | 44 | 21.2 |
| Unknown | 39 | 1.8 | 0 | 0 | 4 | 1.5 | 7 | 2.5 | 24 | 1.8 | 0 | 0 | 4 | 1.9 |
| ***Pathological*** |  | | | | | | | | | | | | | |
| N0 | 472 | 21.9 | 16 | 28.6 | 65 | 24.0 | 68 | 24.7 | 289 | 21.6 | 4 | 33.3 | 30 | 14.4 |
| N1 | 161 | 7.5 | 7 | 12.5 | 26 | 9.6 | 29 | 10.5 | 86 | 6.4 | 0 | 0 | 13 | 6.3 |
| Unknown | 1524 | 70.7 | 33 | 58.9 | 180 | 66.4 | 178 | 64.7 | 960 | 71.9 | 8 | 66.7 | 165 | 79.3 |
| **Metastatic disease** | | | | | | | | | | | | | | |
| Present | 429 | 19.9 | 11 | 19.6 | 74 | 27.3 | 60 | 21.8 | 247 | 18.5 | 3 | 25.0 | 34 | 16.3 |
| Absent | 1728 | 80.1 | 45 | 80.4 | 197 | 72.7 | 215 | 78.2 | 1088 | 81.5 | 9 | 75.0 | 174 | 83.7 |
| **Primary site** | | | | | | | | | | | | | | |
| Favorable | 827 | 38.3 | 19 | 33.9 | 86 | 31.7 | 106 | 38.5 | 502 | 37.6 | 2 | 16.7 | 112 | 53.8 |
| Unfavorable | 1327 | 61.5 | 37 | 66.1 | 185 | 68.3 | 168 | 61.1 | 831 | 62.2 | 10 | 83.3 | 96 | 46.2 |
| Unknown | 3 | 0.1 | 0 | 0 | 0 | 0 | 1 | 0.4 | 2 | 0.1 | 0 | 0 | 0 | 0 |
| **Metastatic site** | | | | | | | | | | | | | | |
| Lungs | 166 | 19.7 | 6 | 3.6 | 27 | 16.3 | 21 | 12.7 | 101 | 60.8 | 2 | 1.2 | 9 | 5.4 |
| Bone or bone marrow | 233 | 27.7 | 5 | 2.1 | 42 | 18 | 33 | 14.2 | 135 | 57.9 | 2 | 0.9 | 16 | 6.9 |
| Distant lymph nodes | 160 | 19.0 | 5 | 3.1 | 26 | 16.3 | 25 | 15.6 | 89 | 55.6 | 2 | 1.3 | 13 | 8.1 |
| Soft tissue | 105 | 12.5 | 7 | 6.7 | 20 | 19 | 15 | 14.3 | 58 | 55.2 | 1 | 1 | 4 | 3.8 |
| Other | 178 | 21.1 | 5 | 2.8 | 23 | 12.9 | 21 | 11.8 | 109 | 61.2 | 3 | 1.7 | 17 | 9.6 |
| ***FOXO1* fusion status** | | | | | | | | | | | | | | |
| + | 409 | 19 | 12 | 21.4 | 62 | 22.9 | 61 | 22.2 | 228 | 17.1 | 1 | 8.3 | 45 | 21.6 |
| - | 192 | 8.9 | 6 | 10.7 | 31 | 11.4 | 16 | 5.8 | 117 | 8.8 | 1 | 8.3 | 21 | 10.1 |
| Unknown | 1556 | 72.1 | 38 | 67.9 | 178 | 65.7 | 198 | 72.0 | 990 | 74.2 | 10 | 83.3 | 142 | 68.3 |
| ***PAX fusion partner (for fusion +)*** | | | | | | | | | | | | | | |
| PAX3 | 289 | 70.7 | 6 | 50.0 | 43 | 69.4 | 48 | 78.7 | 161 | 70.6 | 0 | 0 | 31 | 68.9 |
| PAX7 | 64 | 15.6 | 6 | 50.0 | 6 | 9.7 | 7 | 11.5 | 34 | 14.9 | 0 | 0 | 11 | 24.4 |
| Unknown | 56 | 13.7 | 0 | 0 | 13 | 21.0 | 6 | 9.8 | 33 | 14.5 | 1 | 100 | 3 | 6.7 |
| **Radiation therapy** | | | | | | | | | | | | | | |
| Yes | 1594 | 73.9 | 42 | 75.0 | 206 | 76.0 | 202 | 73.5 | 988 | 74.0 | 8 | 66.7 | 148 | 71.2 |
| No | 562 | 26.1 | 14 | 25.0 | 65 | 24.0 | 73 | 26.5 | 346 | 25.9 | 4 | 33.3 | 60 | 28.8 |
| Unknown | 1 | 0 | 0 | 0 | 0 | 0 | 0 | 0 | 1 | 0.1 | 0 | 0 | 0 | 0 |
| **Study number** | | | | | | | | | | | | | | |
| D9602 | 392 | 18.2 | 5 | 8.9 | 38 | 14.0 | 49 | 17.8 | 225 | 16.9 | 1 | 8.3 | 74 | 35.6 |
| D9802 | 108 | 5 | 3 | 5.4 | 17 | 6.3 | 16 | 5.8 | 61 | 4.6 | 1 | 8.3 | 10 | 4.8 |
| D9803 | 597 | 27.7 | 16 | 28.6 | 70 | 25.8 | 69 | 25.1 | 394 | 29.5 | 2 | 16.7 | 46 | 22.1 |
| ARST0331 | 338 | 15.7 | 11 | 19.6 | 39 | 14.4 | 44 | 16.0 | 220 | 16.5 | 2 | 16.7 | 22 | 10.6 |
| ARST0531 | 448 | 20.8 | 5 | 8.9 | 17 | 6.3 | 16 | 5.8 | 60 | 4.5 | 0 | 0 | 9 | 4.3 |
| ARST08P1 | 167 | 7.7 | 13 | 23.2 | 58 | 21.4 | 60 | 21.8 | 278 | 20.8 | 4 | 33.3 | 35 | 16.8 |
| ARST0431 | 107 | 5 | 3 | 5.4 | 32 | 11.8 | 21 | 7.6 | 97 | 7.3 | 2 | 16.7 | 12 | 5.8 |

RMS: Rhabdomyosarcoma; NOS: Not otherwise specified; BRMS: Botryoid rhabdomyosarcoma; IRS: Intergroup Rhabdomyosarcoma Study

**Supplemental Table S2:** Racial distribution of the Hispanic cohort

| **Hispanic Cohort** | | **American Indian or Alaska Native** | | **Asian** | | **Black** | | **Native Hawaiian or other Pacific Islander** | | **White** | | **Unknown** | |
| --- | --- | --- | --- | --- | --- | --- | --- | --- | --- | --- | --- | --- | --- |
| N | % | N | % | N | % | N | % | N | % | N | % | N | % |
| 271 | 100 | 2 | 0.7 | 2 | 0.7 | 6 | 2.2 | 1 | 0.4 | 169 | 62.4 | 91 | 33.6 |

**Supplemental Table S3:** Race/ethnicity of patients in the corresponding SEER cohort

| **Characteristic** | **Entire Cohort** | | **Race/Ethnicity** | | | | | | | | | | | |
| --- | --- | --- | --- | --- | --- | --- | --- | --- | --- | --- | --- | --- | --- | --- |
|  |  | | **Asian or Pacific Islander** | | **Hispanic** | | **Non-Hispanic Black** | | **Non-Hispanic White** | | **American Indian or Alaskan Native** | | **Unknown** | |
|  | **N** | **%** | **N** | **%** | **N** | **%** | **N** | **%** | **N** | **%** | **N** | **%** | **N** | **%** |
| Overall No. | 1242 | 100 | 111 | 8.9 | 299 | 24.1 | 196 | 15.8 | 615 | 49.5 | 16 | 1.3 | 5 | 0.4 |

**Supplemental Table S4:** Comparison of race/ethnicity distribution between COG cohort and SEER cohort

| **Cohort** | **Race/Ethnicity** | | | | | | |
| --- | --- | --- | --- | --- | --- | --- | --- |
|  | **Hispanic** | **Non-Hispanic Asian or Pacific Islander or American Indian/Alaska Native^1^** | **Non-Hispanic Black** | **Non-Hispanic Unknown** | **Non-Hispanic White** | **Total** | P-value* |
| **COG** | 271 | 68 | 275 | 60 | 1335 | 2009^2^ | <0.0001 |
| **SEER** | 299 | 127 | 196 | 5 | 615 | 1242 |  |
| **Total** | 570 | 195 | 471 | 65 | 1950 | 3251 |  |

COG: Children's Oncology Group, SEER: Surveillance, Epidemiology, and End Results

^1^ Non-Hispanic Asian, Pacific Islander or American Indian/Alaska Native were combined for comparison

^2^ 148 Unknown race were removed from COG dataset

* P-value was obtained using Chi-squared test

**Supplemental Table S5:** Analysis of effect of race/ethnicity on treatment for RMS

| **Characteristic** | **Race/Ethnicity** | | | | | | | | P-value* |
| --- | --- | --- | --- | --- | --- | --- | --- | --- | --- |
|  | **Asian** | | **Hispanic** | | **Non-Hispanic Black** | | **Non-Hispanic White** | |  |
|  | **N** | **%** | **N** | **%** | **N** | **%** | **N** | **%** |  |
| **IRS group** | | | | | | | | | 0.303 |
| I | 11 | 4.0 | 36 | 13.2 | 39 | 14.3 | 187 | 68.5 |  |
| II | 3 | 1.0 | 32 | 10.7 | 43 | 14.4 | 220 | 73.8 |  |
| III | 31 | 3.2 | 129 | 13.3 | 131 | 13.5 | 679 | 70.0 |  |
| **Radiation therapy** | | | | | | | | | 0.903 |
| yes | 42 | 2.9 | 206 | 14.3 | 202 | 14.0 | 988 | 68.7 |  |
| no | 14 | 2.8 | 65 | 13.1 | 73 | 14.7 | 346 | 69.5 |  |

IRS: Intergroup Rhabdomyosarcoma Study

* P-value was obtained using Chi-squared test

1. Other includes American Indian or Alaska Native, Native Hawaiian or other Pacific Islander [↑](#footnote-ref-1)
